# Necrological

**Published:** 1881-03

**Authors:** 


### NECROLOGICAL.

" Death is the crown of life:
Were death denied, poor man would live in vain."

*Office of Recording Secretary Georgia Medical Society, Savannah, Georgia, January 24th,* 1881.

It is the painful duty of this Committee to report to your Society the death of our fellow member, DR. HABERSHAM, who has just been taken away from us in the prime of manhood: he whom for so many years we were accustomed to see sitting with us at our weekly gathering; he whom we have honored by selecting him for our President, is gone and his place will know him no more. His quiet and Christian-like life was marked by a great devotion to the duties of his profession, and by his unremitting efforts to elevate the standard of medical ethics. Sensitive on all points of honor, he scorned and manfully discountenanced any degrading or dubious action, and, though entitled by birth and by a thorough medical education to be in the foremost ranks, he moved unostentatiously among his acquaintances and his patients, and has left in their recollection a pleasing and lasting impression.

He had the affection of a large circle of family and friends, and the depth of affliction caused by his death can only be estimated by the monument of affectionate devotion that crowned his last sufferings.

DR. JOSEPH CLAY HABERSHAM, son of Dr. J. C. Habersham, grandson of Major John Habersham, of the Continental army, and great-grandson of the Governor James Habersham, one of the first settlers in this State, was born in Savannah, Oct. 9th, 1829. He studied his profession in Harvard Medical College, where he graduated in 1853. Returning to his native home, he entered immediately the field of professional usefulness. In 1857, he married Miss Mary Ann Stiles, daughter of Joseph Stiles, Esq., of Savannah. In 1861, during the civil war, he offered his services to the Confederate Government; was made a full Surgeon, and in that capacity held various posts of trust and importance. At the termination of the contest he again resumed the duties of his profession and has been actively and successfully engaged until his last illness. In 1866; he was elected Vice President of the Medical Association of the State of Georgia. He was elected President of this Society in 1876.

In 1870 the city authorities selected him to fill the important office, of Health Officer, which position he held until 1875.

In 1854, 1858 and 1876, he remained faithful at his post, and was ever to be found ready to heed and alleviate the cry of his afflicted fellow citizens in the direful scourges which decimated our city during those summers. In 1878 he was again selected to fill the position of Health Officer, and held the office until 1880.

*Resolved*, That while we bow with submission to the decree of Providence, it is with sincere grief; and that in our hearts we will ever cherish with kindly affection and esteem the memory of our departed brother.

*Resolved*, That a page in our book of minutes be set apart to his memory and inscribed with his name.

*Resolved*, That a copy of this report and resolutions be sent to his bereaved family, together with our heartfelt condolence; and the same be published.

J. C. LeHardy, M. D.
Chairman.
R. P. Myers, M. D.
T. J. Charlton, M. D.

Attest: J. Weichselbaum, M. D., Rec. Secretary.

*War Department, Surgeon-General's Office, Washington, February* 25, 1881.

The death of GEO. A. OTIS, Surgeon and Brevet Lieutenant-Colonel, U. S. Army.—It is with profound regret and a sense of loss, not only to this corps, but to the medical profession, that the death of George Alexander Otis, Surgeon and Brevet Lieutenant-Colonel, U. S. Army, is announced to the medical corps of the army.

Born at Boston, Massachusetts, November 12, 1830, he graduated with the degrees of A. B. and A. M. from Princeton College; entered the Medical Department of the University of Pennsylvania, and received his degree of M. D. from that institution in 1850; visited Europe, and prosecuted his studies in London and Paris, and returning to this country he established himself at Springfield, Mass.; appointed Surgeon 27th Massachusetts Volunteers, September, 1861, he held this position until appointed Surgeon U. S. Volunteers, August 30, 1864. After the close of the war he entered the Medical Corps, U. S. Army, as Assistant Surgeon, February 28, 1866; became Captain and Assistant Surgeon, July 28, 1866; Major and Surgeon, March 17, 1880, having received the four brevets of

Lieutenant-Colonel of Volunteers, Captain, Major, and Lieutenant-Colonel, U. S. Army, for meritorious services during the war. While Surgeon of the 27th Massachusetts Volunteers he served in Virginia, North and South Carolina, and was on special duty in charge of the hospital steamer Cosmopolitan, in the Department of the South. Assigned to duty in this office July 22, 1864, he was curator of the Army Medical Museum, and in charge of the Division of Surgical Records until his death.

He was editor of the Richmond *Medical Journal* for three years, member of the leading medical societies of America, and corresponding member of various similar societies in Europe, and a contributor to prominent medical journals. Surgeon Otis, with his personal observations of the surgical collections abroad, brought indefatigable industry and untiring energy to the development of the surgical and anatomical collections of the Army Medical Museum, which he has made the most valuable of their kind in the world. The compilation of the "Surgical Volumes of the Medical and Surgical History of the War" has placed Surgeon Otis confessedly among the most prominent contributors to surgical history.

While on duty in this office Surgeon Otis wrote for publication no less than ten reports on subjects connected with military surgery, etc.; among which are his most valuable and exhaustive reports on "Excision of the Head of the Femur for Gunshot Injury," and on "Amputation at the Hip-joint in Military Surgery." Of great culture, retentive memory, and a remarkable facility of expression, he was as a compiler and writer, conscientious in his analyses, giving his deductions from the facts before him with modesty, but decision. With such a record it is needless to speak of his zeal, his ambition, or his devotion to his profession, and especially to the reputation of the corps of which he was so bright an ornament. While devoting himself to the preparation of the third and last volume (now more than half completed) of the "Medical and Surgical History of the War," he died in this city February 23, 1881. His untimely death will be deeply deplored, not only by the medical corps of the army, but by the whole medical profession at home and abroad.

Joseph K. Barnes, Surgeon-General.

